# *Phyllanthus muellerianus* (Euphorbiaceae) Restores Ovarian Functions in Letrozole-Induced Polycystic Ovarian Syndrome in Rats

**DOI:** 10.1155/2019/2965821

**Published:** 2019-05-14

**Authors:** Eveline Christiane Ndeingang, Patrick Brice Defo Deeh, Pierre Watcho, Albert Kamanyi

**Affiliations:** Animal Physiology and Phytopharmacology Laboratory, University of Dschang, P.O. Box 67, Dschang, Cameroon

## Abstract

Polycystic ovarian syndrome (PCOS) is one of the common causes or female infertility.* Phyllanthus muellerianus* (Euphorbiaceae) is a plant used to treat various ailments including frequent menstruation and anovulation. We investigated the effects of* P. muellerianus* extracts on estrus cyclicity, lipid profile, oxidative stress-related markers, sex hormones, and ovarian architecture in letrozole-induced PCOS in rats. After induction of PCOS using letrozole (1 mg/kg/day), normal (n=6), and PCOS (n=108; distributed into 18 groups of 6 animals/group) rats were treated orally for 7 or 14 days with distilled water (10 ml/kg/day), clomiphene citrate (2 mg/kg/day), metformin (500 mg/kg/day), and aqueous or methanolic extract of* P. muellerianus* (30, 60, and 120 mg/kg). Estrus cyclicity, body, and sexual organ (ovaries and uterus) weights, biochemical and histological parameters were measured. There were letrozole-induced PCOS characterized by irregular estrus cyclicity, elevated (p<0.05-0.01) glycaemia, ovarian weight, triglycerides, total cholesterol, LDL cholesterol, VLDL cholesterol, malondialdehyde, luteinizing hormone (LH), and testosterone concentrations, but there were low (p<0.05-0.001) HDL cholesterol, estradiol, progesterone, catalase, peroxidase, and superoxide dismutase levels, compared with control. PCOS rats had multiple cysts compared with control. These reproductive, biochemical, and structural alterations were alleviated by* P. muellerianus* extracts. For instance,* P. muellerianus* restored the estrus cyclicity with a remarkable effect after 14 days of treatment. Moreover,* P. muellerianus* significantly decreased (p<0.001) LH and testosterone (both extracts; 30, 60, and 120 mg/kg) levels, but increased (p<0.01) estradiol (aqueous extract; 60 mg/kg) concentration. Cystic follicles were also decreased after plant application.* P. muellerianus* alleviated reproductive, hormonal, and structural alterations in PCOS rats. This plant could be useful in the management/treatment of reproductive and metabolic disorders related to PCOS.

## 1. Introduction

Polycystic ovary syndrome (PCOS), the most prevalent hormonal disorders among women of reproductive age is a heterogeneous endocrine and metabolic disorder, causing irregular menstrual cycles, dyslipidemia, excessive body weight, oxidative stress, hyperandrogenism, and infertility [1, 2]. PCOS affects 5 to 10% of reproductive-aged women and 40% of affected women experience infertility, making this condition the leading cause of anovulatory infertility [3]. In women with PCOS, the normal ovarian function is disturbed mainly by hyperandrogenism and elevated level of luteinizing hormone (LH) [4], thus resulting in multiple cysts [5]. PCOS increased gonadotropin-releasing hormone (GnRH) pulse frequency, which favors LH production over follicle stimulating hormone (FSH) [6]. This increase in LH concentration subsequently promotes androgens production in the theca cells, while the relative FSH deficiency reduces the ability of granulosa cells to convert androgen into estrogen and impairs follicle maturation and ovulation [7].

Oxidative stress (OS) is also linked to a higher risk of infertility in patients with PCOS. Many studies have shown that OS-related biochemical parameters such as malondialdehyde (MDA), glutathione peroxidase (GPx), catalase, and superoxide dismutase (SOD) are abnormal in patients with PCOS [8]. Moreover, OS is associated with overproduction of reactive oxygen species (ROS), which could cause DNA damage and mutations in tumor suppressor genes, leading to uncontrolled ovarian cells proliferation, development of multiple cysts, and infertility [9].

Letrozole is an aromatase inhibitor commonly used for the treatment of breast cancer, but it is associated with metabolic and reproductive disorders. Indeed, the inhibition of aromatase by letrozole decreases the conversion of androgens to estrogens, leading to an accumulation of androgens in the ovary [10]. Previous reports have shown that letrozole is efficient in establishing PCOS in rats [11, 12]. These animals developed many characteristics of human PCOS, including abnormal follicles [11], hyperglycemia [13], oxidative stress [9], and altered sex hormones (testosterone, estrogens, LH and FSH) levels [11].

The therapeutic treatment of PCOS involved the use of several drugs such as metformin and clomiphene citrate, but they are commonly associated with serious side effects. A new therapeutic approach with fewer side effects, easy availability, and broad spectrum is required. Previous studies reported the efficacy of some plants such as* Trigonella foenum-graecum* [2],* Ecklonia cava* [14] and* Allium fistulosum* [12] in the restoration of ovarian function in PCOS rats.* Phyllanthus muellerianus* (Kuntze) Exell (Euphorbiaceae) commonly called “Mbolongo” in eastern Cameroon, is a tropical plant extensively used for the treatment of digestive disorders, frequent menstruation and anovulation [15, 16]. Phytochemical screening of the barks and leaves of this plant revealed the presence of various components such as gallic acid, isoquercitrin, caffeic acid, geraniin, furosin, corilagin, astragalin, rutin, phaselic acid, methyl gallate, chlorogenic acid, and 3,5-o-dicaffeoylquinic [17]. Moreover,* P. muellerianus* possesses antihyperglycemic [18], antihyperlipidemic [19], antioxidant [20], and aphrodisiac [21] properties in normal rats, but its action in PCOS rats is unknown. Considering the above-mentioned pharmacological properties,* P. muellerianus* may be a good candidate for the treatment of PCOS-related metabolic and reproductive disorders. Therefore, the present study was undertaken to investigate the effects of aqueous and methanolic extracts of* P. muellerianus* on estrus cyclicity, blood glucose level, lipid profile, oxidative stress-related biochemical parameters, sex hormones, and ovarian histology in letrozole-induced PCOS in rats.

## 2. Materials and Methods

### 2.1. Plant Material and Preparation of Aqueous and Methanolic Extracts

The fresh roots of* P. muellerianus* were collected in April 2016 in Tonga, West Region of Cameroon. Botanical identification was done at the Cameroon National Herbarium in Yaoundé-Cameroon (voucher specimen N° BWPV03). Roots were cut, shade-dried for 7 days and powdered with an electric mixer.

The powder of* P. muellerianus* (250 g) was infused in boiling water (1500 ml) for 15 minutes and filtered. The filtrate obtained was dried in an oven (55°C) for 48 hours. At the end, 21.15 g of aqueous extract of* P. muellerianus *were obtained (extraction yield: 11.82%).

The methanolic extract of* P. muellerianus *was obtained by macerating 2500 g of powder in methanol (5000 ml) for 72 hours. The mixture was filtered and evaporated using a rotary evaporator (75°C) under reduced pressure. After evaporation, 26.68 g of methanolic extract was obtained (extraction yield: 9.37%).

### 2.2. Animals

Adult female Wistar rats (180-200 g) were obtained from the animal house of the Animal Biology Department, Faculty of Science of the University of Dschang, Cameroon. The animals were maintained at room temperature (22-23°C) with a reverse natural light-dark cycle (about 12 h of light and 12 h of dark cycle) and free access to a standard diet and water. Only rats with at least three consecutive regular appearance of estrus stages in order (estrus cyclicity) were used in the study. Throughout the treatment period, animals were weighed (twice a week) and vaginal smear was observed under microscope in order to identify the estrus stage. The project was presented and validated by the scientific committee of the Department of Animal Biology, University of Dschang (Date, 05.06.2015), which follows the internationally accepted standard ethical guidelines for laboratory animal use and care as described in the European Economic Community guidelines; Directive 86/609/EEC, of the 24th November 1986 [22].

### 2.3. Animal Grouping and Induction of PCOS

Females with regular estrus cyclicity as described above were selected and distributed into 2 sets. The first set (control group, n=6) was administered orally with vehicle (0.9% NaCl solution) once daily. The second set (n=152) was orally treated for 21 days with letrozole (1 mg/kg/day) dissolved in 0.9% NaCl to induce PCOS. Vaginal smears were collected daily and examined microscopically for the identification of estrus stage. At the end of the induction period, rats of the first set and 6 rats of the second set were randomly selected, sacrificed by cervical dislocation under anesthesia (diazepam: 10 mg/kg and ketamine: 50 mg/kg). Biochemical and histological examinations were performed to confirm PCOS in rats. PCOS rats exhibited the main features of PCOS such as hyperglycemia, hyperandrogenism, and multiple cysts as reported previously [23]. Additionally, irregular estrus cyclicity (rats having a disturbed appearance of the four estrus stages) was the main criteria to select PCOS rats [23]. Subsequently, 108 PCOS rats were distributed into 18 groups (6 rats per group) and treated orally for 7 or 14 days with distilled water (10 ml/kg/day), clomiphene citrate (2 mg/kg/day), metformin (500 mg/kg/day), and aqueous or methanolic extract of* P. muellerianus* (30, 60, and 120 mg/kg/day). A healthy control group (n=6) was administered with distilled water (10 ml/kg/day) for 14 days. The effective dose of letrozole (1 mg/kg) and treatment period (21 days) were chosen from reference studies [11, 24]. Doses of* P. muellerianus* (30, 60 and 120 mg/kg/day) were selected from our pilot study (unpublished data). During the treatment period, vaginal smears were collected daily and examined microscopically for the identification of estrus stage. After 7 and 14 days of treatment, animals were sacrificed and the body and organ (ovaries and uterus) weights, blood glucose level, lipid profile, oxidative stress-related biochemical parameters, sex hormones, and ovarian histology were evaluated.

### 2.4. Estrus Cycle Motoring

Vaginal smears were collected daily (8-10 a.m.) to determine the reproductive cycle of each animal. As described in our previous study [25], the predominance of nucleated epithelial cells was classified as proestrus (the first stage). The estrus (the second stage) was characterized by the presence of cornified squamous epithelial cells which occur in clusters. Metestrus (the third stage) is a mix of cell types with a predominance of leukocytes and a few nucleated and/or cornified squamous epithelial cells. Diestrus (the fourth stage) consists predominantly of leukocytes [26].

### 2.5. Oral Glucose Tolerance Test

This test was done before and after induction of PCOS, and after treatments with the reference drugs and plant extracts. Rats were fasted for 6 hours and glycemia was determined in a tail blood sample using a handheld glucometer (Accucheck-active, Roche Diagnostics) before (time 0) a single oral administration of glucose (2.5 g/kg) and at 30, 60, 90 and 120 minutes after administration [27].

### 2.6. Sacrifice and Sample Collection

After 7 or 14 days of treatment, rats were sacrificed by cervical dislocation under diazepam (10 mg/kg) and ketamine (50 mg/kg) anesthesia, and abdominal artery blood was collected into heparinized tubes. Plasma was obtained by centrifugation (3000 g for 10 min) and stored at -20°C until assayed for lipid profile, oxidative stress parameters, and sex hormones. After blood collection, ovaries and uterus were removed and weighed and histological study was done.

### 2.7. Lipid Profile, Oxidative Stress Parameters and Sex Hormones

The lipid profile, including plasmatic total cholesterol (TC), low density lipoprotein cholesterol (LDL-C), very low density lipoprotein cholesterol (VHDL-C), high-density lipoprotein cholesterol (HDL-C), and triglycerides (TG), was evaluated using standard colorimetric kits (CORMAY, Łomianki, POLAND) as described previously [28, 29]. The LDL-C and VLDL-C levels were calculated based on Friedewald's equation: LDL = TC – TG/5 – HDL; VLDL = TG/5 [28].

Plasmatic estradiol, LH, FSH, progesterone, and testosterone levels were measured using commercial kits as described previously [30, 31].

Malondialdehyde (MDA) level was estimated as described by Olszewska-Słonina et al. [32]. Total peroxidase contents were measured as described by Giustarini et al. [33]. Superoxides dismutase (SOD) and catalase activities were determined as described by Serra et al. [34] and Hadwan [35], respectively.

### 2.8. Histopathological Evaluation

Ovaries were processed step by step through 10% neutral formalin fixation (24 h), paraffin embedding, and longitudinally and serially sectioned at 4 *μ*m with a microtome. The samples were stained with hematoxylin and eosin and assessed microscopically according to the methods described by Kafali et al. [11]. Preantral, antral, atretic and cystic follicles, and corpus luteum were identified [36].

### 2.9. Statistical Analysis

All data were expressed as mean ± SEM (standard error of the mean). ANOVA for repeated measures followed by Tukey HSD posttest was used for multiple comparisons. Statistical analysis was done using STATISTICA software Version 8.0 (StatSoft, Inc., Tulsa, USA). The significance of the difference was fixed at p<0.05.

## 3. Results

### 3.1. Effects of Letrozole on Estrus Cyclicity, Blood Glucose Level, Body and Ovarian Weights, and Sex Hormones after 21 Days of Treatment

**Figure 1:**
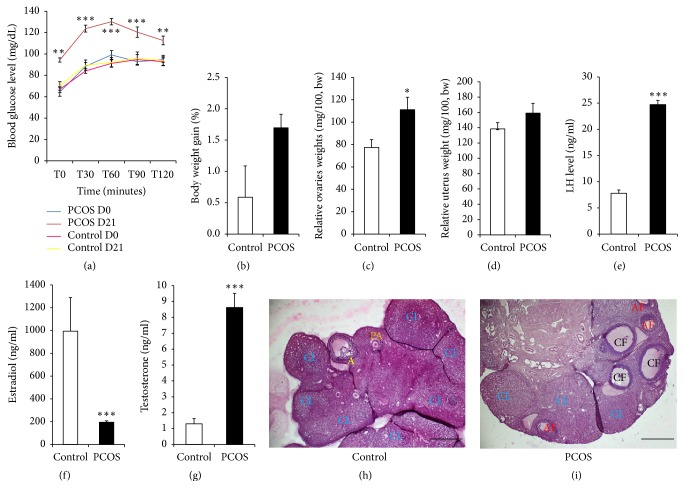
*Letrozole treatment of female rats resulted in reproductive and metabolic features of PCOS*. (a) Blood glucose tolerance test; (b) body weight gain; (c, d) relative ovary and uterus weights, respectively; (e-g) ovarian LH, estradiol, and testosterone levels, respectively. Values are means ± SEM, number of animals per group =6. *∗*p<0.05; *∗∗*p<0.01; *∗∗∗*p<0.001: significantly different compared with control. (h) Section of ovary from a control rat showing growing follicles (at different stages) and a large number of corpus luteum. (i) Section of ovary from PCOS rats exhibiting many cystic and atretic follicles and decreased corpus luteum. CL: corpus luteum; CF: cystic follicle; AF: atretic follicle; PA: preantral follicle; A: antral follicle. Magnification x40. Calibration bar =100 *μ*m.

On day 0 (before letrozole treatment), all rats had a regular estrus cyclicity. After 5, 10, 15, and 21 days of oral treatment with letrozole, 57, 59, 72, and 46% of rats had an irregular cyclicity, respectively. In the control group, all rats had a regular estrus cycle throughout the study period (Table 1). PCOS rats showed elevated blood glucose level (p<0.01-0.001), compared with control (Figure 1(a)). No significant change in body weight was observed in both groups (Figure 1(b)). In PCOS rats, the relative ovary weight was significantly increased (p<0.05), but no significant change in uterus weight was observed compared to the control group (Figures 1(c) and 1(d)). Letrozole-induced PCOS rats showed an elevated (p<0.001) testosterone and LH concentrations, with reduced (p<0.001) estradiol level (Figures 1(e)–1(g)). Moreover, section of ovary from control rats showed growing follicles (at different stages) and a large number of corpus luteum (Figure 1(h)) while those from PCOS rats exhibited many cystic and atretic follicles and decreased corpus luteum (Figure 1(i)). Overall, these features confirmed PCOS in rats after 21 days of letrozole treatment.

### 3.2. Effects of Different Treatments on Body Weight and Relative Weights of the Ovaries and Uterus

**Figure 2:**
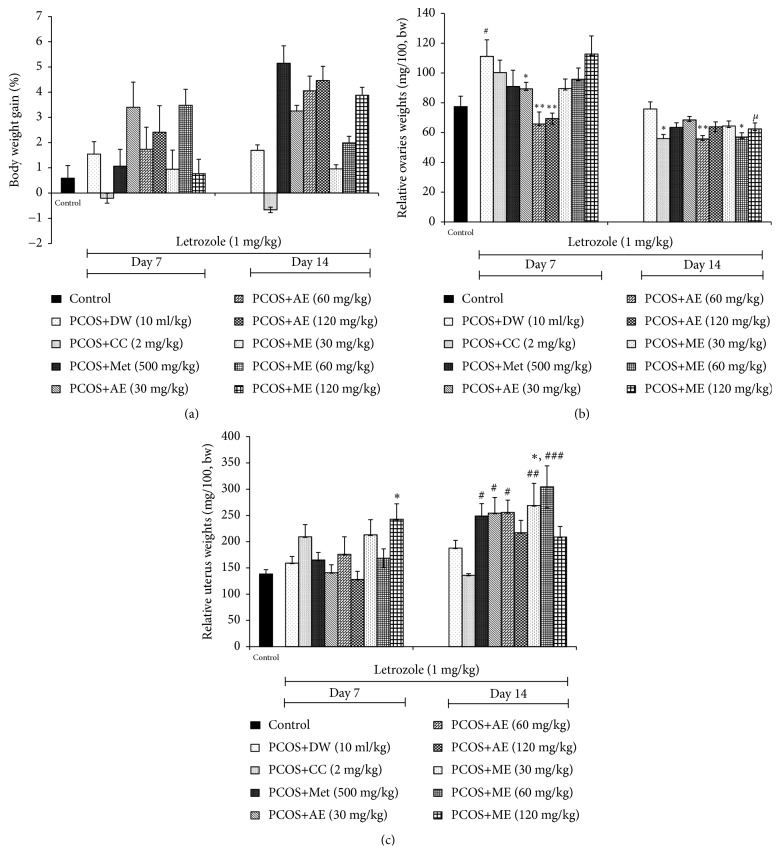
*Effects of clomiphene citrate, metformin, aqueous and methanolic extracts of P. mullerianus on body (a), ovaries (b) and uterus (c) weights after 7 and 14 days of treatment*. Values are means ± SEM, number of animals per group = 6. DW: distilled water; CC: clomiphene citrate; Met: metformin; AE: aqueous extract; ME: methanolic extract; *∗*p<0.05; *∗∗*p<0.05; *∗∗∗*p<0.05: significantly different compared with control. ^#^p<0.05; ^##^p<0.01; ^###^p<0.001: significantly different compared with PCOS group. ^*μ*^p<0.01: significantly different compared with day 7 for the same group.

There was no change in the body weight of rats administered for 7 and 14 days with reference products or plant extracts, compared with control (Figure 2(a)).

In general, the relative weights of the ovaries were reduced in all groups (except control group) after 14 days of treatment compared to those treated for 7 days. A significant decrease in ovarian weight was noticed in animals treated for 7 days with the aqueous extract of* P. muellerianus* (30 mg/kg: p<0.05; 60 mg/kg: p<0.01) compared to the SOPK group. After 14 days of treatment, clomiphene citrate, and aqueous or methanolic extract of* P. muellerianus* significantly (p<0.05-0.01) decreased the ovarian weight (Figure 2(b)). Moreover, in rats treated for 14 days with the methanolic extract of* P. muellerianus *at 120 mg/kg, a significant decrease (p<0.05) in ovarian weight was noted, compared with rats treated for 7 days with the same dose (Figure 2(b)).

The methanolic extract of* P. muellerianus* (120 mg/kg) significantly increased (p <0.05) the uterus weight after 7 days of treatment, compared with control. After 14 days of treatment, metformin, aqueous, and methanolic extracts of* P. muellerianus* (30 and 60 mg/kg) significantly (p<0.05-0.01) elevated the uterus weight, compared with SOPK group. The highest value was observed in rats administered for 14 days with the methanolic extract of* P. muellerianus* at 60 mg/kg (Figure 2(c)).

### 3.3. Effects of Different Treatments on Estrus Cyclicity

In the control group, all rats had a regular estrus cyclicity while all untreated PCOS rats had an irregular estrus cycle. Remarkably, the aqueous (120 mg/kg: 66.67%) and methanolic (30 mg/kg: 66.67%; 60 mg/kg: 55.56%) extracts of* P. muellerianus *increased the percentage of rats with regular estrus cyclicity compared with PCOS group (Table 2).

### 3.4. Effects of Different Treatments on Blood Glucose Tolerance Test

Blood glucose levels were significantly elevated (p<0.05-0.001) in untreated PCOS rats (0, 90 and 120 min), compared with control. In all PCOS groups (except rats treated with metformin for 14 days), blood glucose levels at 30, 60, 90, and 120 min were elevated, compared to their respective basal values (before glucose administration). Interestingly, metformin (120 min), aqueous (60 mg/kg, 90 and 120 min), and methanolic (30 mg/kg, 120 min; 60 mg/kg, 30, 90 and 120 min) extracts of* P. mullerianus* significantly decreased (p<0.05-0.001) blood glucose level after 14 days of treatment, compared with PCOS group (Table 3).

### 3.5. Effects of Different Treatments on Lipid Profile

#### 3.5.1. Effects on Triglycerides

**Figure 3:**
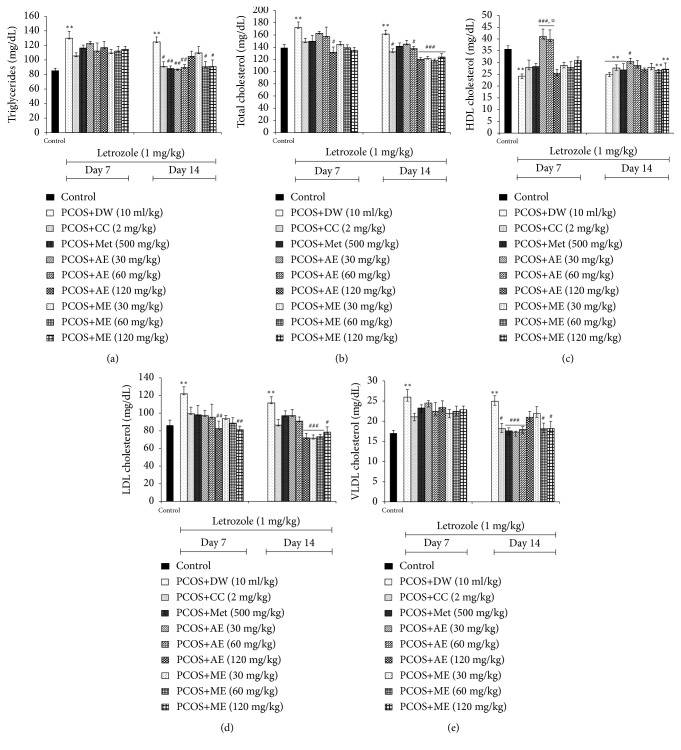
*Effects of clomiphene citrate, metformin, aqueous and methanolic extracts of P. mullerianus on triglycerides (a), total cholesterol (b), HDL cholesterol (c), LDL cholesterol (d), and VLDL cholesterol (e) after 7 and 14 days of treatment*. Values are means ± SEM, number of animals per group = 6. DW: distilled water; CC: clomiphene citrate; Met: metformin; AE: aqueous extract; ME: Methanolic extract; *∗*p<0.05; *∗∗*p<0.05; *∗∗∗*p<0.05: significantly different compared with control. ^#^p<0.05; ^##^p<0.01; ^###^p<0.001: significantly different compared with PCOS group.

TG level was significantly elevated (p<0.01) in untreated SOPK group, compared with control. In all SOPK-treated groups, no significant change in TG level was observed after 7 days of treatment. On the contrary, clomiphene citrate, metformin, and aqueous (30 and 60 mg/kg) or methanolic (60 and 120 mg/kg) extract of* P. muellerianus *significantly decreased (p<0.05-0.01) the TG level, compared to the untreated SOPK group (Figure 3(a)).

#### 3.5.2. Effects on Total Cholesterol

Elevated (p<0.01) TC level was noticed after SOPK induction. However, a decrease (p<0.05) in TC level was observed after 7 days of* P. mullerianus *(aqueous extract at 120 mg/kg) application. Clomiphene citrate, aqueous (120 mg/kg), and methanolic (30, 60 and 120 mg/kg) extracts of* P. muellerianus *significantly lowered (p<0.05-0.001) the TC level after 14 days of treatment, compared with untreated SOPK group. The efficacy of the methanolic extract was more pronounced (Figure 3(b)).

#### 3.5.3. Effects on HDL Cholesterol

The installation of SOPK was associated with a significant reduction (p<0.01) in ovarian HDL cholesterol level. However, it was significantly increased (p<0.001) 7 days after plant (aqueous extract, 30 and 60 mg/kg) administration. In all groups (except aqueous extract, 30 mg/kg) treated for 14 days with reference products or plant extracts, the HDL cholesterol level remained lower than that of control group. Interestingly, the aqueous extract of* P. muellerianus* (60 mg/kg) significantly increased (p<0.05) the HDL cholesterol level after 14 days of treatment, compared with SOPK group (Figure 3(c)).

#### 3.5.4. Effects on LDL and VLDL Cholesterol

Letrozole treatment significantly increased (p<0.01) ovarian LDL and VLDL cholesterol levels, compared with control. LDL cholesterol concentration was significantly reduced (p<0.01) in rats administered for 7 days with aqueous and methanolic extracts of* P. muellerianus* (120 mg/kg), but no change (p>0.05) in VLDL cholesterol level was observed in all treated groups. After 14 days of treatment, the ovarian LDL cholesterol level was significantly lowered (p<0.05-0.001) in rats administered with aqueous (120 mg/kg) or methanolic (30, 60, and 120 mg/kg) extract of* P. muellerianus, *compared with SOPK group. VLDL cholesterol level was also reduced in rats administered for 14 days with clomiphene citrate (p<0.05), metformin (p<0.001), and aqueous (30, 60 mg/kg; p<0.001) or methanolic (60, 120 mg/kg; p<0.05) extract of* P. muellerianus *(Figures 3(d) and 3(e)).

### 3.6. Effects of Different Treatments on Oxidative Stress Parameters

#### 3.6.1. Effects on MDA Level

**Figure 4:**
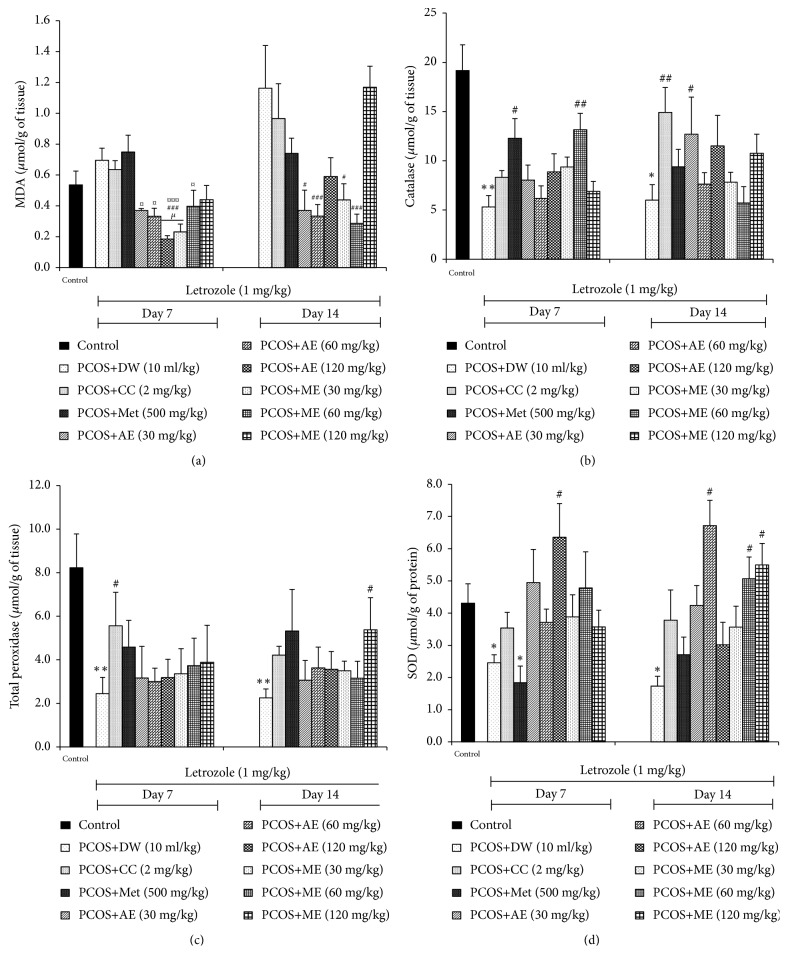
*Effects of clomiphene citrate, metformin, aqueous and methanolic extracts of P. mullerianus on malondialdehyde (a), catalase (b), total peroxidase (c) and superoxide dismutase (d) levels after 7 and 14 days of treatment*. Values are means ± SEM, number of animals per group = 6. DW: distilled water; CC: clomiphene citrate; Met: metformin; AE: aqueous extract; ME: methanolic extract; *∗*p<0.05; *∗∗*p<0.05: significantly different compared with control. ^#^p<0.05; ^##^p<0.01; ^###^p<0.001: significantly different compared with PCOS group. ^¤^P <0.05; ^¤¤¤^P <0.001: significantly different compared with metformin group.

Compared with the untreated SOPK group, we found that MDA level was significantly decreased (p<0.05-0.001) in rats administered with aqueous (30, 60 and 120 mg/kg) or methanolic (30 and 60 mg/kg) extract of* P. muellerianus* after 7 and 14 days of treatment (Figure 4(a)).

#### 3.6.2. Effects on Catalase Activity

Catalase activity was significantly lowered (p<0.01) in rats after SOPK induction. However, females administered for 7 days with metformin and methanolic extract of* P. muellerianus* (60 mg/kg) exhibited high (p<0.05-0.01) catalase activity in the ovaries. After 14 days of treatment, catalase activity was significantly elevated in rats treated with clomiphene citrate (p<0.01) and aqueous extract of* P. muellerianus* (30 mg/kg; p<0.05), compared to the SOPK group (Figure 4(b)).

#### 3.6.3. Effects on Total Peroxidases and SOD Activities

Ovarian total peroxidases (p<0.01) and SOD (p<0.05) activities were also decreased after SOPK induction. Remarkably, clomiphene citrate and aqueous extract of* P. muellerianus* (120 mg/kg) significantly elevated (p<0.05) total peroxidases and SOD activities, respectively, after 7 days of treatment. After 14 days of treatment, high total peroxidases activities (p<0.05) were noticed in rats administered with methanolic extract of* P. muellerianus* (120 mg/kg), compared to the SOPK group. Similarly, SOD activity was elevated (p<0.05) in rats treated for 14 days with aqueous (60 mg/kg) and methanolic (60 and 120 mg/kg) extracts of* P. muellerianus* (Figures 4(c) and 4(d)).

### 3.7. Effects of Different Treatments on Sex Hormones

After induction of PCOS, LH and testosterone levels were significantly increased while progesterone and estradiol concentrations were decreased compared with control.

Metformin and* P. muellerianus *(both extracts at all doses) significantly lowered (p < 0.001) LH level after 7 days of treatment. All rats (except those administered with aqueous extract, 30 mg/kg) treated for 14 days with plant extracts also had a low (p < 0.001) LH level, compared with PCOS group. In all groups, FSH concentrations were statistically unchanged after treatments. On the contrary, progesterone levels remained low in rats administered for 7 days with aqueous (120 mg/kg) and methanolic (30 and 120 mg/kg) extracts of* P. muellerianus, *compared with control. Remarkably, a significant decrease (p < 0.05-0.001) in progesterone level was observed in rats treated with metformin (7 and 14 days), aqueous (7 days: 30 and 60 mg/kg; 14 days: 60 mg/kg), and methanolic (14 days: 60 mg/kg) extracts of* P. muellerianus, *compared with PCOS group. When compared with PCOS group, estradiol level was significantly increased (p < 0.05-0.001) in rats administered with clomiphene citrate (7 and 14 days), metformin (7 days), and aqueous extract of* P. muellerianus *(7 and 14 days: 60 mg/kg). Metformin, aqueous and methanolic extracts of* P. muellerianus* (60 and 120 mg/kg) significantly reduced (p < 0.05-0.01) the testosterone level after 7 days of treatment. Testosterone concentration was also lowered in rats treated for 14 days with metformin and* P. muellerianus *(both extracts at all doses) (Table 4).

### 3.8. Effects of Different Treatments on Ovarian Histology

**Figure 5:**
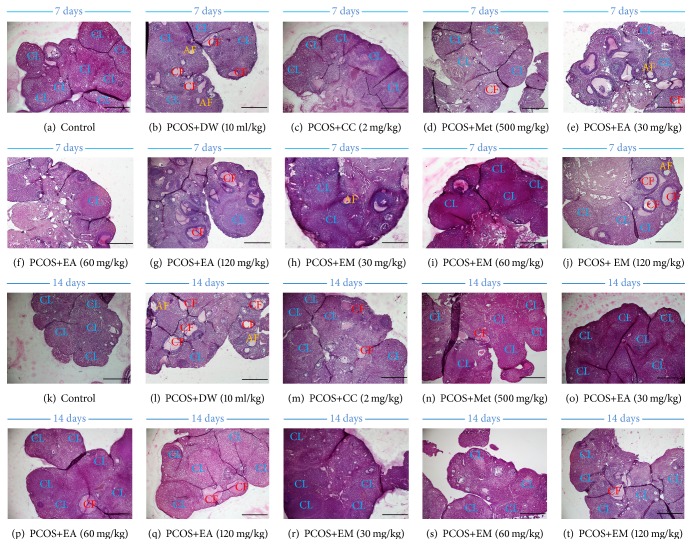
*Effects of clomiphene citrate, metformin, aqueous and methanolic extracts of P. mullerianus on ovarian histology after 7 and 14 days of treatment*. DW: distilled water; CC: clomiphene citrate; Met: metformin; AE: aqueous extract; ME: methanolic extract; CL: corpus luteum; CF: cystic follicle; AF: atretic follicle; PA: preantral follicle; A: antral follicle. Magnification x40. Calibration bar =100 *μ*m.

Letrozole treatment negatively affected the ovarian architecture by decreasing the number of corpus luteum and increasing cystic and atretic follicles (Figures 5(a), 5(b), 5(k), and 5(l)). Clomiphene citrate (Figures 5(c) and 5(m)), metformin (Figures 5(d) and 5(n)), and aqueous (Figures 5(e), 5(f), 5(g), 5(o), 5(p), and 5(q)) and methanolic (Figures 5(h), 5(i), 5(j), 5(r), 5(s), and 5(t)) extracts of* P. muellerianus* alleviated these detrimental effects of letrozole after 7 and 14 days of treatment. The efficacy of the plant extracts was more pronounced after 14 days of treatment.

## 4. Discussion

This study clearly showed that aqueous and methanolic extracts of* P. muellerianus *alleviated the reproductive and metabolic disorders in PCOS rats after 7 and 14 days of treatment. This therapeutic effect was characterized by the restoration of estrus cyclicity, the reduction of blood glucose level and oxidative stress as well as the improvement of lipid profile and sex hormones. The histological study of ovarian tissues also showed significant improvement in ovarian architecture (reduction of cystic follicles and elevated number of corpus luteum) after plant application, thus corroborating our findings that* P. muellerianus *is a potential agent for the treatment of reproductive and metabolic disorders related to PCOS.

The efficacy of letrozole (an aromatase inhibitor) in establishing PCOS in rats is well documented [11, 12]. It acts by inhibition of aromatase, leading to low conversion of androgens to estrogens, resulting in an excessive accumulation of androgens in the ovary [10]. The hormonal changes negatively affect or stop the maturation of follicles, leading to anovulation [37]. Letrozole-induced PCOS model is a good method because animals developed many characteristics of human PCOS, including hyperandrogenism and abnormal follicles [11], hyperglycemia [13], and oxidative stress [9]. As expected, we observed in the current study that letrozole easily induced PCOS in rats after 21 days of continuous administration. These findings are similar to other reports [11, 14, 38].

The estrus cycle is negatively affected in rats with PCOS, mainly due to the alteration of steroid hormones, which regulate ovarian function [39]. In the present study, PCOS rats had irregular estrus cyclicity whereas control rats exhibited a regular one at the end of the induction period. Similarly, Rajan et al. [38] and Yang et al. [14] reported that letrozole-induced PCOS in rats is associated with prolonged estrus cycle. Interestingly, 44.44% of PCOS rats administered with clomiphene citrate had a regular estrus cycle after 14 days of treatment. This result is evident because clomiphene citrate is a first-line treatment for patients with PCOS. Treatment of PCOS rats with aqueous or methanolic extract of* P. muellerianus *restored the estrous cyclicity possibly by modulating the aromatization of androgens into estrogen, by lowering LH level, by improving circulating estradiol concentration and inducing ovulation.

Hyperglycemia is also considered as an important indicator of PCOS. The blood glucose level was significantly elevated in PCOS rats, compared with control. The insulin resistance and low glucose tolerance created by letrozole are mainly due to elevated androgen concentrations as reported by Desai et al. [40]. The effectiveness of metformin in the treatment of anovulatory infertility among patients with PCOS has been proven [41]. As reported in the literature [42], we observed in the current study that metformin-treated rats displayed a significantly decrease in blood glucose level, compared with untreated PCOS rats. Remarkably,* P. muellerianus* also decreased the glucose levels after treatment. We could therefore suggest that* P. muellerianus* reduces glucose resistance by controlling glucose homeostasis, improving insulin secretion and potentiating the insulin-mediated uptake of glucose.

PCOS has been linked to dyslipidemia. In untreated PCOS group, a significant reduction in HDL cholesterol, but elevated triglycerides, total cholesterol, LDL cholesterol and VLDL cholesterol were recorded, compared with control. These parameters were significantly improved after* P. muellerianus* application. These findings are similar to those of Romualdi et al. [43] and Rafraf et al. [44] on soy isoflavone and chamomile tea extract, respectively. The hyperlipidemia observed in untreated PCOS rats was also correlated with an upward trend in body weight and a significant increase in ovarian weight which could be due to the anabolic properties of letrozole, associated with fat accumulation and multiple cysts formation in the ovary, respectively [45]. The significant reduction in ovarian weight and elevated uterus weight in PCOS rats treated with plant extracts could indicate normal follicle formation and uterotonic effects of* P. muellerianus. *However, further studies are required to clarify these effects.

It has been reported that the antioxidant enzymes decline in patients with PCOS [46]. In the present study, notable changes in oxidative stress parameters were displayed in PCOS group. In this group, a significant increase in MDA level, but reduction in catalase, total peroxidases and SOD activities were noticed, compared with control. Jahan et al. [42] also demonstrated that letrozole-induced PCOS in rats is associated with oxidative stress in the ovaries. In the ovaries of PCOS rats, the oxidative stress-related biochemical parameters were significantly improved by* P. muellerianus. *Similarly, the efficacy of* Phoenix dactylifera*,* Clonorchis sinensis*,* Mentha piperita,* and* Thymus vulgaris* commonly used for the treatment of PCOS symptoms is linked to their antioxidant potentials and their ability to improve sex hormones and restore ovary function [46].

Measurement of sex hormone levels (especially testosterone, LH, and estradiol) is recommended for diagnosing PCOS [47]. Indeed, elevated serum testosterone and LH concentrations and low estradiol, progesterone, and FSH levels are the most consistent hormonal features to diagnose PCOS in woman [47]. In this study, letrozole-induced PCOS rats showed high LH and testosterone levels, but low estradiol and FSH concentrations, compared with control. These results matched those of some researchers [48, 49] and further confirm the PCOS condition. Of great interest,* P. muellerianus* treatment alleviated hyperandrogenism in rats as evidenced by significant decreased testosterone and LH levels after 7 and 14 days of oral administration, which could promote follicular development and induce ovulation. In parallel,* Ecklonia cava* extract improves sex hormones in PCOS rats by significantly upregulating FSH concentration and downregulating testosterone and LH levels [14]. Moreover, Bardei [50] demonstrated that rasp berry fruit extract induced a significant reduction in testosterone level and restored ovarian function in PCOS rats, due to its antioxidant potential.* Vitex agnus-castus* fruit extract also exhibited an antiandrogenic effect in PCOS rats by increasing aromatase process (increasing the conversion of testosterone into estradiol) leading to low testosterone level [51]. Because abnormal increased in testosterone level contributes to the pathogenesis of PCOS [11], the downregulation of this hormone after* P. muellerianus* treatment may have beneficial effects on reproductive disorders in PCOS. As observed in the current study, a decline in estrogen level in untreated PCOS rats is correlated with multiple cysts formation and low number of corpus luteum in the ovary [52]. However,* P. muellerianus* improved estradiol level after treatment. Since the abnormal increase in estradiol level is correlated with multiple cysts formation, abnormal estrus cyclicity and anovulation [53], the improvement in estradiol level after treatment with* P. muellerianus* may suggest a beneficial effect on ovarian function. The improvement in LH, estradiol, and testosterone levels by metformin corroborated previous studies which demonstrated that this drug improves ovarian-related markers and induces ovulation in mice with PCOS [54, 55].

PCOS has been reported to be associated with ovarian damage [42]. The ovary sections of PCOS rats revealed many cystic and atretic follicles and low number of corpus luteum [42, 56]. Moreover, the size of cystic follicles was larger than that of other follicles which can be correlated to the increase levels of intraovarian androgens [57]. Thickness of granulosa layer was lowered while that of theca layer was increased in different follicles of untreated PCOS rats. These detrimental effects of letrozole on ovary architecture were corrected by* P. muellerianus* extracts, probably due to its antioxidant and antiandrogenic properties.

**Figure 6:**
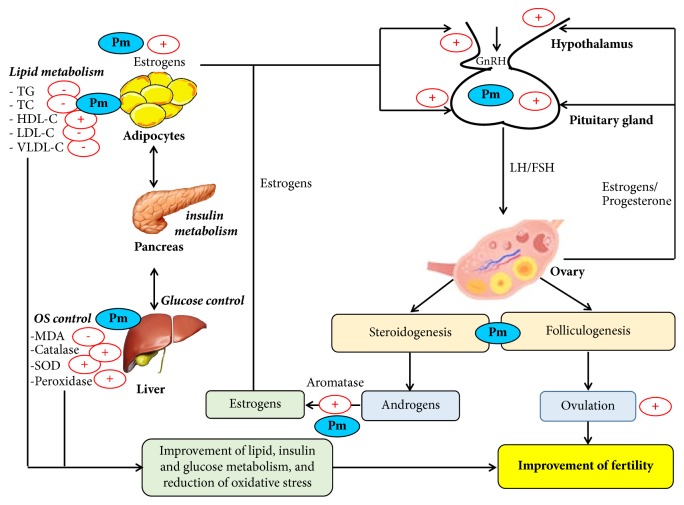
*Proposed mechanism of action of P. mullerianus.* Pm:* Phyllanthus muellerianus*; TG: triglycerides; TC: total cholesterol, HDL-C: high-density lipoprotein cholesterol; LDL-C: low density lipoprotein cholesterol; VLDL-C: very low density lipoprotein cholesterol; MDA: malondialdehyde; SOD: superoxide dismutase; GnRH: gonadotropin-releasing hormone; LH: luteinizing hormone; FSH: follicle stimulating hormone. + = stimulation; - = inhibition.

To the best of our knowledge, the present study is the first to illustrate the beneficial effect of* P. muellerianus* in PCOS rats. Based on the results obtained, it could be proposed that* P. muellerianus* acts by (1) modulating the pulsatile release of GnRH, LH, and FSH, (2) amplifying the aromatization of androgens into estrogens, and (3) stimulating estrogens production by adipocytes, responsible for the restoration of estrus cyclicity and ovulation induction. Additionally, the beneficial effects of* P. muellerianus *could be mediated through its antihyperglycemic and antioxidant potentials (Figure 6). However, more mechanistic studies (transcription factor for instance) are needed to further support this mechanism.

## 5. Conclusion

The present study confirmed that letrozole-induced PCOS in rats is associated with reproductive and metabolic disorders.* P. muellerianus* restores estrus cyclicity, reduces blood glucose level and oxidative stress, improves lipid profile and sex hormones, and prevents ovarian damage in PCOS rats after 7 and 14 days of treatment. This plant might be considered as an alternative therapeutic remedy to treat reproductive and metabolic disorders in patients with PCOS.

## Figures and Tables

**Table 1:** Effects of 21 days of treatment with letrozole on the percentage of irregular estrus cycle.

Groups	Duration of treatment				
	D0	D1-5	D6-10	D11-15	D16-21
	Percentage of irregular estrus cycle (%)				
PCOS	0	57	59	72	46
Control	0	0	0	0	0

Number of rats per group=6. PCOS: polycystic ovarian syndrome. D: day.

**Table 2:** Percentage of PCOS rats with regular estrus cycle after treatments with clomiphene citrate, metformin, and aqueous and methanolic extracts of *P. mullerianus* for 14 days.

	Control	PCOS+DW	PCOS+CC	PCOS+MET	PCOS+AE	PCOS+AE	PCOS+AE	PCOS+ME	PCOS+ME	PCOS+ME
		(10 ml/kg)	(2 mg/kg)	(500 mg/kg)	(30 mg/kg)	(60 mg/kg)	(120 mg/kg)	(30 mg/kg)	(60 mg/kg)	(120 mg/kg)
D0-D4	100	0	0	0	0	22.22	0	22.22	0	0
D5-D9	100	0	33.33	22.22	22.22	0	44.44	11.11	44.44	33.33
D10-D14	100	0	44.44	33.33	22.22	33.33	66.67	66.67	55.56	44.44

Number of rats per group = 6. D: day; DW: distilled water; CC: clomiphene citrate; MET: metformin; AE: aqueous extract; ME: methanolic extract; PCOS: polycystic ovarian syndrome.

**Table 3:** Effects of clomiphene citrate, metformin, and aqueous and methanolic extracts of *P. mullerianus *on glucose tolerance of rats after 7 and 14 days of treatment.

Groups	Doses	Blood glucose level (mg/dL)				
				Time		
		0 min	30 min	60 min	90 min	120 min
Control	10 ml/kg	70.14 ± 3.99	89.57 ± 9.82	92.14 ± 4.55	96 ± 7.88	93.86 ± 4.86

			7 days			

PCOS+Distilled water	10 ml/kg	96.13 ± 3.74*∗*	128.63 ± 2.09^*μμ*^	121 ± 4.65^*μμ*^	137.13 ± 5.56*∗*^, *μμμ*^	131.75 ± 5.09*∗∗*^, *μμμ*^
PCOS+Clomiphene citrate	2 mg/kg	77 ± 2.87	121.29 ± 10.17^*μ*^	125.57 ± 11.84^*μ*^	121.14 ± 9.79^*μ*^	118.43 ± 7.60^*μ*^
PCOS+Metformin	500 mg/kg	76.57 ± 3.21	127.29 ± 4.22^*μμμ*^	125.86 ± 3.28^*μμ*^	122.57 ± 3.56^*μμ*^	126.86 ± 5.47^*μμ*^
PCOS+Aqueous extract	30 mg/kg	75.13 ± 3.30	107.75 ± 7.43^*μμ*^	119.75 ± 4.31^*μμ*^	110.63 ± 4.13^*μμ*^	113.63 ± 3.63^*μμ*^
PCOS+Aqueous extract	60 mg/kg	72.38 ± 4.56	111.25 ± 3.36^*μμ*^	115.13 ± 6.89^*μμμ*^	111.63 ± 4.59^*μμ*^	105.38 ± 3.98^*μ*^
PCOS+Aqueous extract	120 mg/kg	77.38 ± 5.53	106.38 ± 6.09^*μμ*^	118.5 ± 5.15^*μμ*^	109.63 ± 4.83^*μμ*^	119.25 ± 3.95^*μμ*^
PCOS+Methanolic extract	30 mg/kg	88.71 ± 4.09	131.14 ± 5.88^*μμμ*^	138.71 ± 4.99^*μμμ*^	134.71 ± 6.14^*μμμ*^	126.71 ± 5.08^*μμ*^
PCOS+Methanolic extract	60 mg/kg	88.43 ± 2.58	143.43 ± 3.30^*μμμ*^	140.43 ± 6.44^*μμμ*^	145.43 ± 24.37^*μμμ*^	136 ± 5.27*∗∗*^, *μμ*^
PCOS+Methanolic extract	120 mg/kg	68.57 ± 2.09	129.57 ± 6.79^*μμμ*^	141.57 ± 3.60^*μμμ*^	144.29 ± 8.95^*μμμ*^	127.57 ± 3.33^*μμ*^

			14 days			

PCOS+Distilled water	10 ml/kg	97.86 ± 4.97*∗*	126.14 ± 2.26^*μμ*^	124.29 ± 2.85^*μμ*^	127.29 ± 3.09^*μμ*^	136.86 ± 2.38*∗∗∗*^,*μμμ*^
PCOS+Clomiphene citrate	2 mg/kg	83.83 ± 2.94	112 ± 4.80^*μμ*^	118.83 ± 3.82^*μμ*^	118 ± 7.57^*μμ*^	115.67 ± 2.47^*μμ*^
PCOS+Metformin	500 mg/kg	91.75 ± 5.95	110.13 ± 7.69	114 ± 2.03	124.75 ± 2.33	108.5 ± 8.73^##^
PCOS+Aqueous extract	30 mg/kg	84.88 ± 3.28	110.63 ± 2.28^*μ*^	116.88 ± 5.62^*μ*^	122.38 ± 3.61^*μ*^	119.75 ± 3.96^*μ*^
PCOS+Aqueous extract	60 mg/kg	81.43 ± 2.35	112.71 ± 4.89^*μ*^	111 ± 5.01^*μ*^	103.14 ± 4.31^#,*μ*^	96.57 ± 5.06^###^
PCOS+Aqueous extract	120 mg/kg	79.42 ± 3.74	121.57 ± 3.08^*μ*^	121.71 ± 3.98^*μ*^	123.14 ± 5.28^*μ*^	120.29 ± 3.87^*μ*^
PCOS+Methanolic extract	30 mg/kg	78.25 ± 4.81	108.5 ± 4.39^*μ*^	116.63 ± 7.29^*μ*^	107.75 ± 5.12^*μ*^	97.75 ± 4.22^###^
PCOS+Methanolic extract	60 mg/kg	78.67 ± 5.53	98.33 ± 3.48^#^	117 ± 4.67^*μ*^	103.67 ± 5.81^#, *μ*^	102.5 ± 6.41^##, *μ*^
PCOS+Methanolic extract	120 mg/kg	77.25 ± 2.92	126.38 ± 5.52^*μμ*^	132.13 ± 6.19^*μμμ*^	126.63 ± 3.77^*μμ*^	118 ± 3.85^*μ*^

The values are expressed as mean ± SEM; number of rats per group=6. PCOS: polycystic ovarian syndrome. *∗*p < 0.05; *∗∗*p < 0.01; *∗∗∗*p < 0.001: significantly different compared with control. ^#^p<0.05; ^##^p <0.01; ^###^p <0.001: significantly different compared with PCOS rats treated with distilled water.

^*μ*^p<0.05; ^*μμ*^p <0.01; ^*μμμ*^p <0.001: significantly different compared with the base line value (T0, before glucose administration).

**Table 4:** Effects of clomiphene citrate, metformin, aqueous and methanolic extracts of *P. mullerianus *on plasmatic LH, FSH, progesterone, estradiol, and testosterone levels after 7 and 14 days of treatment.

Groups	Doses	LH	FSH	Progesterone	Estradiol	Testosterone
		(ng/ml)	(ng/ml)	(ng/ml)	(ng/ml)	(ng/ml)
Control	10 ml/kg	7.79 ± 0.64	3.15 ± 0.03	56.15 ± 1.82	995 ± 195.17	1.30 ± 0.33

			7 days			

PCOS+Distilled water	10 ml/kg	24.72 ± 0.81^*∗*^	3.28 ± 0.11	16.65 ± 1.14^*∗∗∗*^	169 ± 20.98^*∗∗∗*^	7.54 ± 1.31^*∗∗∗*^
PCOS+Clomiphene citrate	2 mg/kg	17.98 ± 1.62	3.26 ± 0.03	16.71 ± 1.76^*∗∗∗*^	858.75 ± 45.24^###^	4.95 ± 0.36
PCOS+Metformin	500 mg/kg	13.04 ± 0.83^###^	3.31 ± 0.16	32.86 ± 3.1^#^	645 ± 85.51^###^	1.85 ± 0.33^##^
PCOS+Aqueous extract	30 mg/kg	14.71 ± 1.21^###^	3.34 ± 0.10	37.39 ± 2.44^###^	332.5 ± 20.23	5.59 ± 1.10
PCOS+Aqueous extract	60 mg/kg	11.86 ± 0.94^###^	3.37 ± 0.05	44.56 ± 4.13^###^	648.5 ± 108.98^##^	2.61 ± 0.71^#^
PCOS+Aqueous extract	120 mg/kg	9.58 ± 0.13^###^	3.41 ± 0.02	17.56 ± 2.12^*∗∗∗*^	306.5 ± 12.16	3.16 ± 0.67^#^
PCOS+Methanolic extract	30 mg/kg	9.25 ± 0.32^###^	3.23 ± 0.13	19.82 ± 2.24^*∗∗∗*^	273.5 ± 8.42	6.45 ± 1.30
PCOS+Methanolic extract	60 mg/kg	9.77 ± 0.36^###^	2.97 ± 0.02	29.15 ± 1.09	415.75 ± 38.01	2.17 ± 0.32^##^
PCOS+Methanolic extract	120 mg/kg	11.84 ± 1.34^###^	3.29 ± 0.12	16.65 ± 1.78^*∗∗∗*^	335 ± 21.20	3.15 ± 0.76^#^

			14 days			

PCOS+Distilled water	10 ml/kg	22.62 ± 2.12^*∗*^	2.98 ± 0.05	15.74 ± 2.46^*∗∗∗*^	294.75 ± 12.29^*∗∗∗*^	8.62 ± 0.89^*∗∗∗*^
PCOS+Clomiphene citrate	2 mg/kg	19.71 ± 2.86	3.22 ± 0.18	20.86 ± 1.83	782.5 ± 73.30^##^	4.14 ± 1.57
PCOS+Metformin	500 mg/kg	18.3 ± 2.34	2.93 ± 0.06	38.32 ± 3.59^##^	429 ± 27.99	1.49 ± 0.37^###^
PCOS+Aqueous extract	30 mg/kg	21.76 ± 2.52	3.03 ± 0.07	17.59 ± 1.89^*∗∗∗*^	521 ± 84.79	4.05 ± 1.87^#^
PCOS+Aqueous extract	60 mg/kg	12.58 ± 1.30^###^	3.10 ± 0.05	43.61 ± 4.38^###^	608.5 ± 64.45^##^	1.92 ± 0.27^##^
PCOS+Aqueous extract	120 mg/kg	6.8 ± 1.39^###^	3.13 ± 0.10	30.60 ± 1.39	225.5 ± 35.25	1.53 ± 0.34^###^
PCOS+Methanolic extract	30 mg/kg	9.87 ± 0.29^###^	3.30 ± 0.07	23.87 ± 4.33	191.25 ± 4.87	1.70 ± 0.34^###^
PCOS+Methanolic extract	60 mg/kg	14.17 ± 1.63^###^	2.97 ± 0.11	36.32 ± 3.62^##^	363.75 ± 68.93	1.54 ± 0.36^###^
PCOS+Methanolic extract	120 mg/kg	9.51 ± 0.14^###^	3.05 ± 0.02	30.22 ± 3.96	275 ± 9.78	3.02 ± 0.11^#^

The values are expressed as mean ± SEM; number of rats per group=6. PCOS: polycystic ovarian syndrome. *∗*p < 0.05; *∗∗*p < 0.01; *∗∗∗*p < 0.001: significantly different compared with control. ^#^p<0.05; ^##^p <0.01; ^###^p <0.001: significantly different compared with PCOS rats treated with distilled water.

## Data Availability

The data used to support the findings of this study are available from the corresponding author upon request.

## References

[1] Teede H., Deeks A., Moran L. (2010). Polycystic ovary syndrome: a complex condition with psychological, reproductive and metabolic manifestations that impacts on health across the lifespan. *BMC Medicine*.

[2] Swaroop A., Jaipuriar A. S., Gupta S. K. (2015). Efficacy of a novel fenugreek seed extract (Trigonella foenum-graecum, furocyst™) in polycystic ovary syndrome (PCOS). *International Journal of Medical Sciences*.

[3] Hassanzadeh B. M., Emami S. A., Mousavifar N. (2013). Effects seeds extract on insullin resistance in women with polycystic ovarian syndrome. *Iranian Journal of Pharmaceutical Research*.

[4] Imani B., Eijkemans M. J. C., Te Velde E. R., Fauser B. C. J. M. (2002). A nomogram to predict the probability of live birth after clomiphene citrate induction of ovulation in normogonadotropic oligoamenorrheic infertility. *Fertility and Sterility*.

[5] Franks S., Stark J., Hardy K. (2008). Follicle dynamics and anovulation in polycystic ovary syndrome. *Human Reproduction Update*.

[6] Burt Solorzano C. M., Beller J. P., Abshire M. Y., Collins J. S., McCartney C. R., Marshall J. C. (2012). Neuroendocrine dysfunction in polycystic ovary syndrome. *Steroids*.

[7] McCartney C. R., Eagleson C. A., Marshall J. C. (2002). Regulation of gonadotropin secretion: implications for polycystic ovary syndrome. *Seminars in Reproductive Medicine*.

[8] Murri M., Luque-Ramírez M., Insenser M., Ojeda-Ojeda M., Escobar-Morreale H. F. (2013). Circulating markers of oxidative stress and polycystic ovary syndrome (PCOS): a systematic review and meta-analysis. *Human Reproduction Update*.

[9] Zuo T., Zhu M., Xu W. (2016). Roles of oxidative stress in polycystic ovary syndrome and cancers. *Oxidative Medicine and Cellular Longevity*.

[10] Garcia-Velasco J. A., Moreno L., Pacheco A. (2005). The aromatase inhibitor letrozole increases the concentration of intraovarian androgens and improves in vitro fertilization outcome in low responder patients: a pilot study. *Fertility and Sterility*.

[11] Kafali H., Iriadam M., Ozardali I., Demir N. (2004). Letrozole-induced polycystic ovaries in the rat: a new model for cystic ovarian disease. *Archives of Medical Research*.

[12] Lee Y., Yang H., Lee S., Kwon S., Hong E., Lee H. (2018). Welsh onion root (*Allium fistulosum*) restores ovarian functions from letrozole induced-polycystic ovary syndrome. *Nutrients*.

[13] Zhu J.-P., Teng Y.-C., Zhou J., Lu W., Tao M.-F., Jia W.-P. (2013). Increased mean glucose levels in patients with polycystic ovary syndrome and hyperandrogenemia as determined by continuous glucose monitoring. *Acta Obstetricia et Gynecologica Scandinavica*.

[14] Yang H., Lee S. Y., Lee S. R. (2018). Therapeutic effect of Ecklonia cava extract in letrozole-induced polycystic ovary syndrome rats. *Frontiers in Pharmacology*.

[15] Burkill H. M. (1995). *The Useful Plants of West Tropical Africa*.

[16] Katsayal U. A., Lamal R. S. (2009). Preliminary phytochemical and antibacterial screening of the ethanolic stem bark extract of *Phyllanthus muellerianus*. *Nigerian Journal of Pharmaceutical Sciences*.

[17] Agyare C., Lechtenberg M., Deters A., Petereit F., Hensel A. (2011). Ellagitannins from *Phyllanthus muellerianus* (Kuntze) Exell.: Geraniin and furosin stimulate cellular activity, differentiation and collagen synthesis of human skin keratinocytes and dermal fibroblasts. *Phytomedicine*.

[18] Adeneye A. A. (2012). The leaf and seed aqueous extract of *Phyllanthus amarus* improves insulin resistance diabetes in experimental animal studies. *Journal of Ethnopharmacology*.

[19] Mao X., Wu L.-F., Guo H.-L. (2016). The genus *Phyllanthus*: an ethnopharmacological, phytochemical, and pharmacological review. *Evidence-Based Complementary and Alternative Medicine*.

[20] Boakye Y., Agyare C. (2013). Antimicrobial and antioxidant activities of geraniin and aqueous leaf extract of Phyllanthus muellerianus (Kuntze) Exell. *Planta Medica*.

[21] Ben-Bala K. D., Schmelzer G. H., Gurib-Fakim A. (2008). *Phyllanthus muellerianus* (Kuntze) Exell. *PROTA (Plant Resources of Tropical Africa/Ressources Végétales de L’Afrique Tropicale)*.

[22] EEC (1986). Council Directive 86/609/EEC of 24 November 1986 on the approximation of laws, regulations and administrative provisions of the Member States regarding the protection of animals used for experimental and other scientific purposes. *Offical Journal of the European Communities*.

[23] Ghafurniyan H., Azarnia M., Nabiuni M., Karimzadeh L. (2015). The effect of green tea extract on reproductive improvement in estradiol valerate-induced polycystic ovary polycystic ovarian syndrome in rat. *Iranian Journal of Pharmaceutical Research*.

[24] Rezvanfar M. A., Rezvanfar M. A., Ahmadi A., Shojaei-Saadi H. A., Baeeri M., Abdollahi M. (2012). Molecular mechanisms of a novel selenium-based complementary medicine which confers protection against hyperandrogenism-induced polycystic ovary. *Theriogenology*.

[25] Ngadjui E., Nkeng-Efouet P. A., Nguelefack T. B., Kamanyi A., Watcho P. (2015). High fat diet-induced estrus cycle disruption: effects of Ficus asperifolia. *Journal of Complementary and Integrative Medicine*.

[26] Kulkarni S. K. (1999). *Hand Book of Experimental Pharmacology*.

[27] Fofié C. K., Nguelefack-Mbuyo E. P., Tsabang N., Kamanyi A., Nguelefack T. B. (2018). Hypoglycemic properties of the aqueous extract from the stem bark of ceiba pentandra in dexamethasone-induced insulin resistant rats. *Evidence-Based Complementary and Alternative Medicine*.

[28] Friedewald W. T., Levy R. I., Fredrickson D. S. (1972). Estimation of the concentration of low-density lipoprotein cholesterol in plasma, without use of the preparative ultracentrifuge. *Clinical Chemistry*.

[29] Youmbissi T. J., Djoumessi S., Nouedoui C. (2001). Lipid profile of a group of hypertensive Cameroonians black African. [Profil lipidique d’un groupe d’hypertendus camerounais noirs africains]. *Médecine d'Afrique Noire*.

[30] Ropelato M. G., Rudaz M. C. G., Escobar M. E. (2009). Acute effects of testosterone infusion on the serum luteinizing hormone profile in eumenorrheic and polycystic ovary syndrome adolescents. *The Journal of Clinical Endocrinology & Metabolism*.

[31] Rezvanfar M. A., Rezvanfar M. A., Shahverdi A. R. (2013). Protection of cisplatin-induced spermatotoxicity, DNA damage and chromatin abnormality by selenium nano-particles. *Toxicology and Applied Pharmacology*.

[32] Olszewska-Słonina D. M., Ma̧tewski D., Czajkowski R. (2011). The concentration of thiobarbituric acid reactive substances (TBARS) and paraoxonase activity in blood of patients with osteoarthrosis after endoprosthesis implantation. *Medical Science Monitor*.

[33] Giustarini D., Fanti P., Matteucci E., Rossi R. (2014). Micro-method for the determination of glutathione in human blood. *Journal of Chromatography B*.

[34] Serra J. A., Marschoff E. R., Domínguez R. O. (2000). Comparison of the determination of superoxide dismutase and antioxidant capacity in neurological patients using two different procedures. *Clinica Chimica Acta*.

[35] Hadwan M. H. (2016). New method for assessment of serum catalase activity. *Indian Journal of Science and Technology*.

[36] Gozukara I. O., Pınar N., Ozcan O. (2016). Effect of colchicine on polycystic ovary syndrome: an experimental study. *Archives of Gynecology and Obstetrics*.

[37] Gopal M., Duntley S., Uhles M., Attarian H. (2002). The role of obesity in the increased of obstructive sleep apnea syndrome in patients with polycystic ovarian syndrome. *Sleep Medicine*.

[38] Rajan R. K., Kumar M. S. S., Balaji B. (2017). Soy isoflavones exert beneficial effects on letrozole-induced rat polycystic ovary syndrome (PCOS) model through anti-androgenic mechanism. *Pharmaceutical Biology*.

[39] Sun J., Jin C., Wu H. (2013). Effects of electro-acupuncture on ovarian P450arom, P450c17*α* and mRNA expression induced by letrozole in PCOS rats. *PLoS ONE*.

[40] Desai N. R., Shrank W. H., Fischer M. A. (2012). Patterns of medication initiation in newly diagnosed diabetes mellitus: quality and cost implications. *American Journal of Medicine*.

[41] Johnson N. P. (2014). Metformin use in women with polycystic ovary syndrome. *Annals of Translational Medicine*.

[42] Jahan S., Munir F., Razak S. (2016). Ameliorative effects of rutin against metabolic, biochemical and hormonal disturbances in polycystic ovary syndrome in rats. *Journal of Ovarian Research*.

[43] Romualdi D., Costantini B., Campagna G., Lanzone A., Guido M. (2008). Is there a role for soy isoflavones in the therapeutic approach to polycystic ovary syndrome? Results from a pilot study. *Fertility and Sterility*.

[44] Rafraf M., Zemestani M., Asghari-Jafarabadi M. (2015). Effectiveness of chamomile tea on glycemic control and serum lipid profile in patients with type 2 diabetes. *Journal of Endocrinological Investigation*.

[45] Samy N., Hashim M., Sayed M. (2009). Clinical significance of inflammatory markers in polycystic ovary syndrome: their relationship to insulin resistance and body mass index. *Disease Markers*.

[46] Abasian Z., Rostamzadeh A., Mohammadi M., Hosseini M., Rafieian-kopaei M. (2018). A review on role of medicinal plants in polycystic ovarian syndrome: pathophysiology, neuroendocrine signaling, therapeutic status and future prospects. *Middle East Fertility Society Journal*.

[47] Bednarska S., Siejka A. (2017). The pathogenesis and treatment of polycystic ovary syndrome: what's new?. *Advances in Clinical and Experimental Medicine*.

[48] Orio F., Giallauria F., Palomba S. (2008). Metabolic and cardiopulmonary effects of detraining after a structured exercise training programme in young PCOS women. *Clinical Endocrinology*.

[49] Palomba S., Giallauria F., Falbo A. (2008). Structured exercise training programme versus hypocaloric hyperproteic diet in obese polycystic ovary syndrome patients with anovulatory infertility: a 24-week pilot study. *Human Reproduction*.

[50] Bardei K. (2015). The effects of hydro-alcoholic extract of raspberry fruit on ovarian follicles and serum parameters in poly cystic ovary syndrome-induced rat. *Armaghane Danesh*.

[51] Jelodar G., Askari K. (2012). Effect of Vitex agnus-castus fruits hydroalcoholic extract on sex hormones in rat with induced polycystic ovary syndrome (PCOS). *Physiology and Pharmacology*.

[52] Dewailly D., Robin G., Peigne M., Decanter C., Pigny P., Catteau-Jonard S. (2016). Interactions between androgens, FSH, anti-Mullerian hormone and estradiol during folliculogenesis in the human normal and polycystic ovary. *Human Reproduction Update*.

[53] Diamanti-Kandarakis E., Christakou C., Kandarakis H. (2007). Polycystic ovarian syndrome: the commonest cause of hyperandrogenemia in women as a risk factor for metabolic syndrome. *Minerva Endocrinologica*.

[54] Sander V., Luchetti C. G., Solano M. E. (2006). Role of the N, N′-dimethylbiguanide metformin in the treatment of female prepuberal BALB/c mice hyperandrogenized with dehydroepiandrosterone. *Reproduction*.

[55] Johnson N. (2011). Metformin is a reasonable first-line treatment option for non-obese women with infertility related to anovulatory polycystic ovary syndrome - a meta-analysis of randomised trials. *Australian and New Zealand Journal of Obstetrics and Gynaecology*.

[56] Riley J. C. M., Behrman H. R. (1991). Oxygen radicals and reactive oxygen species in reproduction. *Experimental Biology and Medicine*.

[57] Mahesh V., Mills T., Bagnell C., Mahesh V. B., Dhindsa D. S., Anderson E., Kalra S. P. (1987). Animal models for study of polycystic ovaries and ovarian atresia. *Regulation of Ovarian and Testicular Function*.

